# Tocilizumab-induced hypofibrinogenemia in patients with systemic-onset juvenile idiopathic arthritis

**DOI:** 10.1038/s41598-023-36246-6

**Published:** 2023-06-03

**Authors:** Tingyan He, Jiayun Ling, Jun Yang

**Affiliations:** Department of Rheumatology and Immunology, Shenzhen Children’s Hospital, 7019 Yitian Road, Shenzhen, 518038 China

**Keywords:** Diseases, Rheumatology

## Abstract

Systemic juvenile idiopathic arthritis (SJIA) is a chronic inflammatory disease of childhood with elevated serum IL-6 levels. As an inhibitor of IL-6R, tocilizumab (TCZ) has been approved to treat SJIA patients. TCZ-induced hypofibrinogenemia has been only reported in adult cases and limited small case series with rheumatoid arthritis or giant cell arteritis. Here, we describe the incidence of TCZ-induced hypofibrinogenemia in SJIA patients and its possible influence on bleeding risk. SJIA patients with TCZ treatment in Shenzhen Children’s hospital were retrospectively reviewed. Only those with the data on serum fibrinogen levels were included. Data on clinical manifestations, laboratory parameters, management, and sJADAS10-ESR score were collected. Laboratory data were extracted following the start of TCZ therapy at 2, 4, 8, 12, and 24 weeks thereafter. Seventeen SJIA patients with TCZ treatment were included. Thirteen (76.47%, 13/17) had hypofibrinogenemia. The lowest serum fibrinogen levels were even below 1.5 g/L in seven (41.17%, 7/17) patients. Among four patients without MTX treatment, two had obvious hypofibrinogenemia. Although five patients had already stopped steroid treatment 24 weeks after TCZ treatment, three of them still had hypofibrinogenemia. Only P14 had mild nasal mucosal bleeding occasionally. Coagulation tests were regularly performed in eight patients, of these, six had hypofibrinogenemia, which occurred following one to four doses of TCZ; continuation of TCZ treatment hadn’t further aggravated hypofibrinogenemia. Serum fibrinogen levels were not decreased consistently with the improvement of sJADAS10-ESR score in more than half of these eight patients. Factor XIII was detected in six patients and none was identified with Factor XIII deficiency. TCZ alone may induce hypofibrinogenemia in SJIA patients. Continuation of TCZ treatment may be safe for most SJIA patients. But for SJIA patients with indications of surgery or complicated with MAS, the risk of hemorrhage should be regularly evaluated during TCZ treatment. The association between TCZ-induced hypofibrinogenemia and factor XIII deficiency remains uncertain.

*Trial registration*: Not applicable; this was a retrospective study.

## Introduction

Systemic juvenile idiopathic arthritis (SJIA) is characterized by spiking fever, arthralgia or arthritis, myalgia, evanescent rashes, lymphadenopathy, hepatosplenomegaly, and serositis^1,2^. Overproduction of IL-6 plays an important pathological role in SJIA. Serum IL-6 levels in patients with SJIA correlate with the extent and severity of joint involvement, fever patterns, growth retardation, and osteoporosis^3,4^. Tocilizumab (TCZ), as a recombinant humanized, anti-human interleukin 6 receptors (IL-6R) monoclonal antibody, has been approved for use alone or in combination with disease-modifying anti-rheumatic drugs (DMARDs) to treat patients with SJIA^5,6^.

The most common adverse events of TCZ in the treatment of SJIA include nasopharyngitis, upper respiratory tract infection, and cytopenia^7–9^. Serious adverse events-anaphylactoid reactions and gastrointestinal hemorrhage are relatively rare^7,10^. TCZ had been initially expected to normalize fibrinogen levels. However, TCZ-induced hypofibrinogenemia has been reported recently in adult cases and limited small case series with rheumatoid arthritis (RA) or giant cell arteritis (GCA)^11,12^. TCZ-induced hypofibrinogenemia has never been reported in children with SJIA.

Here, we performed a single, retrospective study of seventeen pediatric patients with SJIA receiving TCZ to assess the incidence rate of TCZ-induced hypofibrinogenemia in these patients and its possible influence on bleeding risk.

## Patients and methods

### Study population and design

SJIA patients with TCZ treatment in Shenzhen Children’s Hospital were retrospectively reviewed from January 2019 to December 2021. Only those with the data on serum fibrinogen levels were included. The study was approved by the ethics committee of Shenzhen Children’s hospital, and written informed consent was obtained from all patients’ legal guardians. SJIA was diagnosed based on the International League of Associations for Rheumatology classification criteria. All patients did not have any novice use of medication except for TCZ, prednisolone, and methotrexate (MTX). During the maintenance treatment period with TCZ, there was no clinical evidence of liver failure or hepatic synthetic dysfunction, disseminated intravascular coagulation, or macrophage activation syndrome (MAS). All experiments were performed following relevant guidelines and regulations.

### Data collection

Clinical data were collected from inpatient or outpatient medical records. Clinical manifestations, laboratory parameters, management, and prognosis were extracted. Laboratory data was extracted following the start of TCZ therapy and at 2 weeks (2W), 4 weeks (4W), 8 weeks (8W), 12 weeks (12W), and 24 weeks (24W) thereafter. Laboratory parameters included total leukocyte count, hemoglobulin, platelet count, erythrocyte sedimentation rate (ESR), C-reactive protein (CRP), serum amyloid A (SAA), serum aminotransferases, serum albumin, ferritin, prothrombin time (PT), international normalized ratio (INR), activated partial thromboplastin time (APTT), and fibrinogen levels. Management included glucocorticoids, TCZ, and MTX. Systemic Juvenile Arthritis Disease Activity Score in 10 joints based on ESR (sJADAS10-ESR) was used for disease activity evaluation^13^.

### Factor XIII activation detection

Factor XIII levels were measured in plasma samples using a semi-quantitative assay by KingMed diagnostics.

### Statistical analysis

Continuous variables were expressed as mean ± SD and categorical data as frequencies. Analysis was performed with GraphPad Prism 8.0 statistical software (GraphPad Software Inc., La Jolla, CA, USA).

### Ethics approval and consent to participate

All participating members were enrolled with the approval of the ethics committee of Shenzhen Children’s Hospital and provided written consent from legal guardians.

## Results

### Clinical manifestations

Seventeen SJIA patients with TCZ treatment were included The initial dosage of intravenous TCZ was 12 mg/kg (weight < 30 kg) or 8 mg/kg (weight ≥ 30 kg) every 2 weeks. Clinical characteristics were summarized in Table 1. The mean onset age was 9.05 years (ranging from 2.17 to 15.42 years). The mean follow-up after initiation of tocilizumab therapy was 15.06 months, ranging from 1 to 24 months. During TCZ treatment, APTT and PT were in normal ranges in all patients. Only P14 had mild nasal mucosal bleeding occasionally. Others had not presented any symptoms of bleeding. No patients had terminated TCZ treatment due to adverse drug reactions or no response.

**Table 1:** Clinical characteristics of patients with systemic-onset juvenile idiopathic arthritis.

P	Onset Age (years)	Sex	At TCZ initiation				24W after TCZ treatment				Follow-up after TCZ (months)	Lowest fib (g/L) during TCZ treatment	Bleeding symptoms during TCZ treatment	XIII deficiency after TCZ
			Prednisone (mg/kg.d)	MTX	Fib (g/L)	sJADAS10score	Prednisone (mg/kg.d)	MTX	Fib (g/L)	sJADAS10score				
P 1	15.42	M	1	15	6.89	34.7	0.14	15	1.73	3	12	1.18	N	Normal
P 2	13	M	1	N	4.86	23.4	0.07	N	2.14	1	6	2.13	N	Normal
P 3	4.25	F	1	10	4.64	40.5	N	10	1.55	3	24	1.25	N	NA
P 4	11.42	M	1	15	6.03	35.7	0.08	15	2.36	1.5	18	1.84	N	NA
P 5	4	M	1.43	12.5	6.02	28.2	0.55	12.5	NA	3.5	24	2.51	N	NA
P 6	10	F	1	N	3.92	19.5	0.23	N	NA	8.5	18	1.15	N	Normal
P 7	13.58	M	N	N	5.47	35	N	N	2.26	1.5	18	2.04	N	NA
P 8	10.67	F	N	15	4.76	23.8	N	15	1.56	0.5	12	1.36	N	NA
P 9	6.92	M	1	12.5	7.99	37.6	0.38	12.5	NA	2.5	24	1.8	N	Normal
P 10	10.83	M	0.75	10	4.37	26.2	0.5	10	NA	6	12	1.84	N	NA
P 11	4	M	2	10	7.46	32.5	0.39	10	NA	4	24	1.36	N	NA
P 12	2.25	F	2	12.5	4.94	34.4	0.34	12.5	1.56	4	12	0.98	N	Normal
P 13	8.42	M	1.67	N	3.32	18	0.07	N	2.03	6	12	1.78	N	Normal
P 14	11	M	1	15	5.38	29	0.53	15	NA	6.5	24	1.53	Mild nasal mucosal bleeding	NA
P 15	2.17	F	2	12.5	7.07	37.3	N	12.5	2.26	3.5	12	1.24	N	NA
P 16	12	F	1.09	15	6.58	29	N	15	2.28	2	8	2.18	N	NA
P 17	14	M	1.62	15	6.92	34.5	0.41	15	NA	18.4	2	1.51	N	NA

*P:* patient, *MTX*: methotrexate, *NA.* not available, *Y*: yes, *N*: no, *M*: male; *F*: female, *TCZ*: tocilizumab, *sJADAS10-ESR* systemic juvenile arthritis disease activity score in 10 joints based on ESR.

### Serum fibrinogen levels

Baseline serum fibrinogen levels were normal or high before TCZ treatment. Serum fibrinogen levels were significantly reduced after TCZ treatment in all patients. Thirteen patients (76.47%, 13/17) had hypofibrinogenemia. The lowest serum fibrinogen levels were even below 1.5 g/L in seven (41.17%, 7/17) patients. Among four patients without MTX treatment, two (P6 and P13) had obvious hypofibrinogenemia. On the contrary, two patients concomitant with MTX showed normal serum fibrinogen levels. Although five patients had already stopped prednisone treatment 24 weeks after initiation of TCZ treatment, three of them still had hypofibrinogenemia (Table 1). The substantial reduction of prednisone didn’t improve hypofibrinogenemia in four patients (P1, P3, P4, and P12) (Table 2). Only P14 had mild nasal mucosal bleeding occasionally.

**Table 2:** Serum fibrinogen levels in SJIA patients following Tocilizumab treatment.

	P 1	P 2	P 3	P 4	P 7	P 8	P 12	P 13
Baseline fib (g/L)	6.89	4.86	4.64	6.03	5.47	4.76	4.94	3.32
Fib at 2W after TCZ (g/L)	2.27	2.3	1.25	1.85	2.22	2.1	1.52	2.13
Fib at 4W after TCZ (g/L)	2.08	2.28	1.47	1.88	2.2	1.54	1.52	2.1
Fib at 8W after TCZ (g/L)	1.96	2.28	1.55	2.06	2.1	1.62	1.57	1.78
Fib at 12W after TCZ (g/L)	1.81	2.13	1.61	1.84	2.36	1.49	1.32	2.15
Fib at 24W after TCZ (g/L)	1.73	2.14	1.55	2.36	2.26	1.56	1.56	2.03

*SJIA* systemic juvenile idiopathic arthritis, *P* patient, *Fib* fibrinogen level, *2W* 2 weeks.

**Figure 1:**
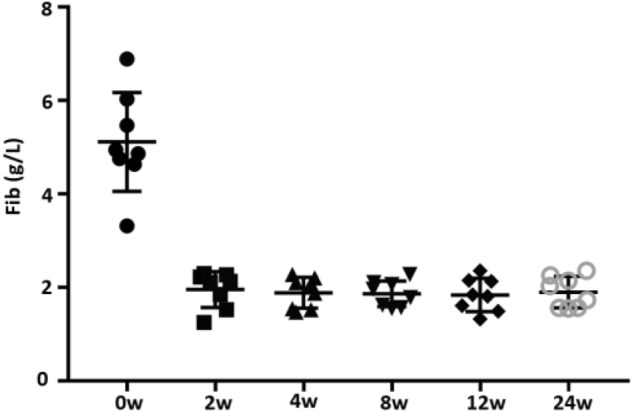
Serum fibrinogen levels were reduced in SJIA patients following TCZ treatment.

**Figure 2:**
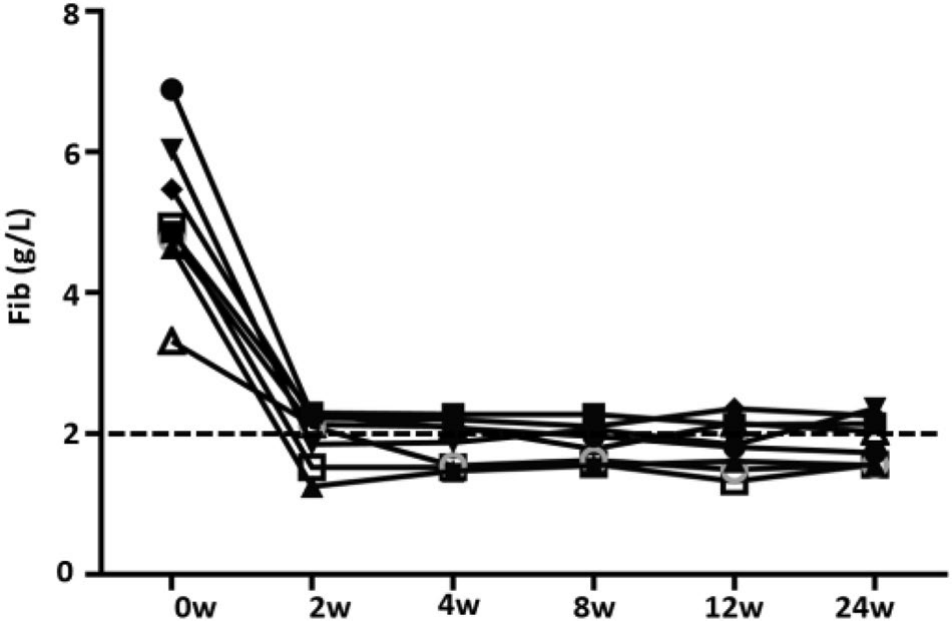
6 of 8 SJIA patients presented with hypofibrinogenemia following one to three doses of TCZ. Continuation of TCZ treatment hadn’t reduced fibrinogen levels progressively.

Coagulation tests were regularly performed in eight patients, of these, six had hypofibrinogenemia, which occurred following one to four doses of TCZ; continuation of TCZ treatment hadn’t further aggravated hypofibrinogenemia (Table 2, Figs. 1, and 2).

### The association of hypofibrinogenemia and sJADAS10-ESR score in SJIA patients

Among the eight patients with regular evaluation of serum fibrinogen levels, hypofibrinogenemia was first observed in four SJIA patients (P3, P4, P12, and P13) with moderate disease activity, one (P1) with minimal disease activity, and one (P8) with inactive disease. Except for two patients (P1 and P2), serum fibrinogen levels were not decreased consistently with the improvement of sJADAS10-ESR score in the other six patients (Tables 2 and 3).

**Table 3:** sJADAS10-ESR score in SJIA patients following Tocilizumab treatment.

sJADAS10-ESR score	P 1	P 2	P 3	P 4	P 7	P 8	P 12	P 13
At TCZ initiation	34.7	23.4	40.5	35.7	35	23.8	34.4	18
2W after TCZ	9.5	7.5	16	9.5	5	5.5	18.5	8.5
4W after TCZ	8	4	12	5.5	5	2.5	8.5	8
8W after TCZ	5.5	2	5	4.5	3.5	1.5	8.5	12
12W after TCZ	4	1	3	1.5	2	0.5	7.5	6
24W after TCZ	3	1	3	1.5	1.5	0.5	4	6

*sJADAS0-ESR* systemic juvenile arthritis disease activity score in 10 joints based on ESR, *SJIA* systemic juvenile idiopathic arthritis, *P* patient, *2W* 2 weeks.

### Factor XIII levels

Based on published literature^14,15^, plasma factor XIII levels were measured by semi-quantitative assay in 6 patients with hypofibrinogenemia. Factor XIII deficiency was not identified in these patients (Table 1).

## Discussion

This study described that hypofibrinogenemia was seen in 76.47% of SJIA patients receiving TCZ treatment. Two patients without MTX treatment had obvious hypofibrinogenemia. On the contrary, two patients concomitant with MTX showed normal serum fibrinogen levels. The reduction or discontinuation of prednisone didn’t improve hypofibrinogenemia. Serum fibrinogen levels were not decreased consistently with the improvement of sJADAS10-ESR score in most patients. Therefore, MTX and prednisone might have negligible or minimal effects on hypofibrinogenemia. TCZ alone may induce hypofibrinogenemia in SJIA patients. Hypofibrinogenemia may not be directly associated with disease activity in SJIA patients treated with TCZ.

Martis et al.^12^ have reported that TCZ-induced hypofibrinogenemia has occurred after an average of eight doses (4–48 doses)^11^. TCZ-induced hypofibrinogenemia occurred after one to four doses of TCZ in most patients in our center. As some cases reported previously, three patients (50%) showed hypofibrinogenemia even after the initial dose. TCZ-induced hypofibrinogenemia may occur earlier in children than in adults. Additional studies are required to explore the role of age in TCZ-induced hypofibrinogenemia.

Around 60% of reported cases have no hypofibrinogenemia-related symptoms. However, some cases may present with ecchymosis, gingival bleeding, and pelvic and inguinal hemorrhage. Imamura et al.^16^ have reported that TCZ treatment may significantly increase the risk of blood loss after total knee arthroplasty by decreasing fibrinogen levels in patients with RA. Except for one patient presenting with mild nasal mucosal bleeding, other SJIA patients with TCZ-induced hypofibrinogenemia in this study have remained asymptomatic in terms of bleeding. Continuation of TCZ hasn’t further reduced fibrinogen levels in all patients or aggravated bleeding symptoms in P14. Considering the possible risk of hypofibrinogenemia induced by TCZ, the risk of hemorrhage should be well assessed before the initiation of TCZ in patients with low platelet counts, other coagulation disorders, and/or macrophage activation syndrome. Surgeons would be better aware of the possible risk of postoperative bleeding complications in patients with TCZ treatment. Therefore, the continuation of TCZ treatment may be safe for most SJIA patients with TCZ-induced hypofibrinogenemia. But for SJIA patients with indications of surgery or complicated with MAS, the risk of hemorrhage should be regularly evaluated during TCZ treatment.

TCZ-induced hypofibrinogenemia has a yet unclear mechanism. The route of administration and the dosage of TCZ has not been proven as risk factors. Fibrinogen is positively regulated by IL-6 and negatively by IL-1 and TNF levels. As an inhibitor of IL-6R, TCZ may inhibit the expression of fibrinogen by hepatocytes. Acquired factor XIII deficiency has been reported in some TCZ-treated patients, showing a correlation between factor XIII B and fibrinogen^14,15^. But more than half of TCZ-induced hypofibrinogenemia cases have normal factor XIII activity^16,17^. As in most reported cases, our patients with TCZ-induced hypofibrinogenemia didn’t have factor XIII deficiency. Further prospective research is required to clarify the association between TCZ-induced hypofibrinogenemia and factor XIII deficiency.

The limitations of this study are the small sample size in a single center and that it is retrospective. Serum fibrinogen level was not regularly detected during the follow-up in all patients. We failed to get enough data to set SJIA patients without TCZ treatment as the control group. Factor XIII activation detection was not performed in all SJIA patients with TCZ-induced hypofibrinogenemia.

## Conclusions

TCZ alone may induce hypofibrinogenemia in SJIA patients. Continuation of TCZ treatment may be safe for most SJIA patients with TCZ-induced hypofibrinogenemia. But for SJIA patients with indications of surgery or complicated with MAS, the risk of hemorrhage should be regularly evaluated during TCZ treatment. Hypofibrinogenemia may not be directly associated with disease activity in SJIA patients treated with TCZ. The association between TCZ-induced hypofibrinogenemia and factor XIII deficiency remains to be further clarified.

## Data Availability

The datasets were collected from the medical records of participating patients in Shenzhen Children’s hospital. All data generated or analyzed during this study are included in this published article. The raw data of this study is available from the corresponding author upon reasonable request.
